# Investigating the association of toll-like receptor 9 rs5743836, rs352140, and rs187084 gene polymorphisms and their mRNA levels with different hepatic fibrosis stages of non-alcoholic fatty liver patients compared to healthy controls

**DOI:** 10.22099/mbrc.2022.43852.1753

**Published:** 2022

**Authors:** Azadeh Rezaie, Meysam Nasiri, Behzad Hatami, Kaveh Baghaie, Hamid Asadzadeh-Aghdaei, Mohammad Reza Zali

**Affiliations:** 1 Gastroenterology and Liver Diseases Research Center, Research Institute for Gastroenterology and Liver Diseases, Shahid Beheshti University of Medical Sciences, Tehran, Iran; 2 Department of Genetics, Damghan Branch, Islamic Azad University, Damghan, Iran.; 3 School of Biology, Damghan University, Damghan, Iran

**Keywords:** Hepatic fibrosis, Non-alcoholic fatty liver, Polymorphism, Toll-like receptor

## Abstract

Recent studies have shown that the level of hepatocyte-derived mitochondrial DNA is elevated in plasma samples obtained from mice and NASH patients, and it has the ability to toll-like receptor 9 (*TLR9*) activation resulting in steatosis, hepatocyte injury, and fibrosis. In this study, we explored the association between *TLR9* rs5743836, rs352140, and rs187084 polymorphism and its plasma mRNA level in non-alcoholic fatty liver (NAFL) patients with different liver fibrosis scores compared to healthy controls. Seventy Iranian patients diagnosed with NAFL, based on fibroscan testing results, were divided into F0-F1 (N=33), F2-F3 (N=19), and F4 (N=18) hepatic fibrosis groups and compared to 22 healthy controls. Genotyping was done using polymerase chain reaction-restriction fragment length polymorphism (PCR-RFLP) and the mRNA expression level of *TLR9* was determined using Real-Time PCR analysis. Results showed no significant association between allelic and genotypic distribution frequency of *TLR9* rs5743836, rs352140, and rs187084 polymorphisms in NAFL patients with hepatic fibrosis compared to healthy controls (P>0.05). However, the mRNA level of *TLR9* was significantly elevated in correlation with hepatic fibrosis progression in NAFL patients compared to healthy controls (P<0.05). As a preliminary study, our data showed a correlative overexpression of *TLR9* mRNA with hepatic fibrosis progression in NAFL patients without the effectiveness of *TLR9* gene polymorphisms.

## INTRODUCTION

Non-alcoholic fatty liver disease (NAFLD) is a wide range of diseases that initiates from the fatty liver and can progress to non-alcoholic steatohepatitis (NASH), liver fibrosis, and finally cirrhosis [1]. NAFLD is the most common form of liver disease, with a 33.9% prevalence in Iran [2]. Its etiology is multifactorial and is associated with dyslipidemia, diabetes mellitus, insulin resistance, and hypertension [1]. An emerging concept is that the accumulation of lipids in hepatocytes leads to the immune system's response, which in turn causes hepatic inflammation as known as NASH [1]. The innate immune system in NASH activates by two possible mechanisms. First, translocate gut microbiota and their products, and activate the innate immune system through Toll-like receptors (TLRs), especially *TLR2*, *TLR4*, and *TLR9* activation, which triggers inflammatory pathways [3-5]. Second, hepatocyte death in the fatty liver is the source of denatured host DNA, which is the TLR-stimulating ligand [3, 6]. 

TLRs are the most important family of pattern recognition receptors; they are the sensors for recognizing bacterial and viral components such as peptidoglycan, bacterial DNA, and lipopolysaccharides [7]. TLRs and adapter molecules (*MyD88*) have an important role in the pathogenesis of NAFLD. TLRs/MyD88 pathway activation creates a signal transduction cascade which results in the release of several chemokines, and cytokines [3, 5]. Animal and human studies have confirmed that proinflammatory cytokines such as TNF-α, IL-1β, IL-6, and chemokines including CCL2, CCL5, and CXCL8 are associated with NASH progression and liver fibrosis [1, 3, 4]. TLR9 is a receptor for unmethylated cytosine phosphate guanine (CpG) containing DNA, the chief component of bacterial and mitochondrial DNA [7]. Lazarus et al. recently analyzed the *TLR9* gene sequence variation and performed exploratory case-control disease association studies for four common TLR9 single-nucleotide polymorphisms (SNPs) [8]. Several data support the role of SNPs in *TLR* genes in modulating the risk of viral and bacterial infection [9]. SNPs may alter promoter activity, gene expression, protein structure, and function, or messenger RNA (mRNA) conformation and stability [10].

In this study, we investigated the association between *TLR9* rs5743836, rs352140, and rs187084 polymorphism and its plasma mRNA level in NAFL patients with different liver fibrosis scores compared to healthy controls.

## MATERIALS AND METHODS

**Subjects: **Seventy Iranian patients referred to Taleghani Hospital in Tehran, Iran, from September 2018 until September 2020, who was diagnosed with NAFL (briefly, patients with fatty liver equal to or more than 5% liver fat on ultrasound, without evidence of liver damage in the form of ballooning of hepatocytes, biochemical evidence of alcohol consumption and chronic liver diseases) were included in this study. NAFLD diagnosis was performed based on AASLD guidelines [1]. Twenty-two sex and age-matched healthy controls (without any history of liver diseases) were enrolled in this study. Based on fibroscan testing results, NAFLD patients were divided into three different groups: patients with mild hepatic fibrosis score (F0-F1) (n=33), patients with advanced hepatic fibrosis score (F2-F3) (n=19), and patients with liver cirrhosis (F4) (n=18). In our study, liver biopsy (as the gold standard for liver fibrosis diagnosis) was not performed because of its limitations and complications for patients. All participants in the study were given written consent, and all study protocols were approved by the Ethics Committee of Shahid Beheshti University of Medical Sciences (ethics code: IR.SBMU.RIGLD. REC.1395.207) based on the Declaration of Helsinki. 

**Liver stiffness measurement: **Liver stiffness was assessed by a trained hepatologist using a transient elastography instrument (FibroScan, Echosens, Paris, France). The M probe was used in all patients, and the results were expressed in kilopascals (kPa). Based on LSM cut-offs described by Castera et al. [11], approved by American Gastroenterological Association, the cut-offs used were: <7.1 kPa (F0-F1, mild fibrosis), 7.1-12.4 (F2-F3, advanced fibrosis), and ≥12.5 kPa (F4; cirrhosis).

**Genotyping of** ***TLR9*** **polymorphisms: **Blood samples were collected from each subject using EDTA-containing tubes. Total RNA and DNA were extracted using the One Step-RNA Reagent (Bio Basic, Canada), according to the manufacturer’s instructions. Genotypes of three common *TLR9* SNPs rs352140, rs5743836, and rs187084 were evaluated using the PCR-restriction fragment length polymorphism (PCR-RFLP) technique. The total volume of PCR reactions was 25 µl with the reaction components consisting of master mix (Ampliqon, Denmark) 12.5 µl, 1 ul each of forward and reverse primers, genomic DNA 5µl (125 ng), and, sterile water 5.5 µl. PCR reaction was carried out using a master cycler^® ^instrument (Eppendorf, Hamburg, Germany). The PCR reaction conditions were as follows: initial denaturation at 95°C for 5min followed by 35 cycles of denaturation at 95°C for 1min; Annealing at 62°C for 45s (for rs352140, and rs187084) and 60°C for 45s (for rs5743836); and extension at 72°C for 1min; with final extension for 5min at 72°C. The primers used for each SNP are listed in Table 1. The PCR product from each gene polymorphism was digested using a specific restriction enzyme as follows: 10ul of PCR product was added to the mixture including each restriction enzyme 1ul (Thermo Scientific), reaction buffer 2 µl, and sterile water 18 µl. The mixture was then incubated at 37°C for 3h and then 65°C for 20min (for *BstU*1 and *BspT*1 respectively) and 80°C for 20min (for *BstN*1). Then the products were analyzed by electrophoresis technique using 2% agarose gel (Invitrogen).

**Table 1:** Summary of Genotyping the *TLR9* gene polymorphisms using PCR-RFLP

Polymorphisms	Primer sequence (5'→3')	Amplicon length (bp)	Restriction enzyme	Fragments size (bp)
rs352140	AGCCACGAAGCTGAAGTTGT GTCAATGGCTCCCAGTTCCT	183	BstU1	GG: 183 AG: 183, 104, 79 AA: 104, 79
rs5743836	GCTGGATGGCCCTGTTGA GCCTCAGGGCCTTGGGAT	123	BstN1	TT: 123 TC: 123,87,36 CC: 87, 36
rs187084	ACTATGGAGCCTGCCTGCCATGATACC ATCCAGCCTTCTTACAAACCTCCCACCC	423	BspT1	CC: 423 TC: 423, 251, 172 TT: 251, 172

**Gene expression assay: **RNA extraction was performed using One Step-RNA Reagent (Bio Basic, Canada) from fresh whole blood according to the manufacturer’s instructions. cDNA synthesized was done using PrimeScript RT Reagent Kit (Takara, Japan). Quantitative mRNA levels of the *TLR9* gene were assessed by a real-time PCR machine, ABI 7500 PCR system (Applied biosciences, USA), using SYBR Green Premix Ex Taq II Kit (Takara, Japan) according to the manufacturer’s instructions. Primers were designed using AllelleID version 7.5 software (premierbiosoft, USA) (Table 2). All primers were designed as intron inclusion, and no genomic DNA amplification was tested for each one. We included 50 ng of each cDNA sample in the PCR reactions (final volume of 10 µL), and the PCR reaction was set as follows: Initial denaturation at 95°C for the 30s followed by 40 cycles of 95°C for 10s and 60°C for 45s. At first, the mRNA *TLR9* expression was normalized by the internal control (*eEF2* gene), and then the mRNA levels in NAFLD patients relative to healthy controls were calculated using the 2^-∆∆Ct^ formula. Briefly, the delta Ct of each patient was subtracted from the average delta Ct in the control group and 2^-∆∆Ct^ was calculated for the patient groups. In order to calculate the deviation value within the healthy control group, the delta Ct of each individual was subtracted from the average delta Ct of that group and 2^-∆∆Ct^ was calculated for each individual. One tube containing no template was included in each run as a negative control. 

**Table 2:** Gene expression primer sequences and amplicons

Gene	Accession number	Primer sequence(5'→3')	Amplicon size (bp)
*TLR9*	NM_017442.3	ATCTCGCAGGCAGTCAAT TGAATGAGTGCTCGTGGTA	103
*eEF2*	NM_001961.4	GCTGATGATGAACAAGATGGA CCGTAGGTGGAGATGATGA	117

**Statistical analyses: **Data are represented as mean ± SEM except where SD is mentioned. Multiple Comparisons between patients with different hepatic fibrosis stages and healthy controls for genotypes frequency distribution were performed using logistic regression analysis. One-way analysis of variance (ANOVA) followed by the Tukey post hoc test was used to compare the *TLR9* mRNA levels between different groups. Correlated gene expression levels with genotype frequency distribution were determined using Pearson correlation analysis. All statistical analyses were done using SPSS version 16 software (IBM, USA), and P<0.05 was considered statistically significant.

## RESULTS AND DISCUSSION

Demographic, laboratory, and clinical data from studied patients have summarized in Table 3. Genotype and allelic frequency of studied *TLR9* gene polymorphisms in patients and healthy controls were shown in Table 4. 

**Table 3:** Patients demographic

**Variable**	**Healthy controls (n=22)**	**F0-F1 (n=33)**	**F2-F3 (n=19)**	**F4 (n=18)**
Age (years)	48.27±10.26	42.03±8.71	49.94±11.65	57.22±10.52
Sex (M/F)	8/14	20/13	11/8	11/7
FBS (mg/dl)	80.68±7.40	99.61±19.02	120.56±35.91	114±34.15
TG (mg/dl)	121.14±47.49	209.5±165.43	159.67±86.41	128±95.47
Total Cholesterol (mg/dl)	164.32±30.59	188.29±45.41	173.29±45.43	144.33±42.84
HDL (mg/dl)	43.86±15.15	49.93±56.18	41.88±12.91	39.40±15.18
LDL (mg/dl)	105.55±18.32	113.86±40.71	102.40±34.69	86.66±34.70
BilT (mg/dl)	0.73±0.42	0.69±0.43	15.37±57.91	1.95±1.38
BilD (mg/dl)	0.26±0.16	0.27±0.23	0.64±1.41	0.69±0.5
AST (U/L)	21.45±10.08	31.69±16.47	32.88±17.78	81.72±126.29
ALT (U/L)	21.77±10.70	53.58±38.86	52.50±41.90	82.44±137.83
ALP (U/L)	147.76±47.52	185.03±52.82	202.44±117.48	269.39±153.91
Plt (10^3^/µl)	236.55±58.18	265.45±73.86	221.07±82.56	120.18±79.81
Ferritin (ng/ml)	101.67±98.96	155.33±139.58	141.14±68.74	253.59±230.42

The frequencies of all alleles and genotypes were in Hardy-Weinberg equilibrium (P>0.05). Our results showed that there was no statistically significant difference in the genotypic and allelic distribution of polymorphisms, rs352140, rs5743836, and rs187084, between patients with hepatic fibrosis compared to healthy controls (P>0.05) (Table 4). 

**Table 4:** Genotypic distribution of TLR9 genetic polymorphisms in NAFLD patients and healthy controls

**Genotypes**		**Healthy controls (%)**	**NAFLD Patients with hepatic fibrosis (%)**	**OR**	**95% CI**	***P***
rs352140	GG	6 (28.6)	21 (30)	1.0	-	-
	GA	11 (52.4)	35 (50)	0.9	0.29-2.82	0.86
	AA	4 (19)	14 (20)	1.0	0.24-4.2	1.0
						
rs574386	TT	16 (72.7)	57 (81.4)	1.0	-	-
	TC	5 (22.7)	13 (18.6)	0.73	0.23-2.35	0.6
	CC	1 (4.5)	0	0	0	-
						
rs187084	TT	10 (47.6)	25 (36.8)	1.0	-	-
	TC	9 (42.9)	29 (42.6)	1.29	0.45-3.67	0.63
	CC	2 (9.5)	14 (20.6)	2.8	0.54-14.6	0.22

**Note:** The sign “-“indicates the reference genotype.

To investigate the relationship between the studied polymorphisms with different degrees of liver fibrosis progression, liver fibrosis patients were randomly divided into three groups: F0-F1, F2-F3, and F4. Then, the genotypic and allelic frequency distribution of each of these polymorphisms in patients with different degrees of liver fibrosis was investigated (Table 5). The results showed that there was no significant difference between the genotypic and allelic frequencies of the studied polymorphisms in patients with F0-F1, F2-F3, and F4 hepatic fibrosis compared to healthy controls (P>0.05) (Table 5). Also, the studied polymorphisms had no significant effect on increasing the risk of developing liver fibrosis from F0-F1 to F2-F3 (P>0.05), F2-F3 to F4 (P>0.05), and F0-F1 to F4 (P>0.05) in NAFLD patients. In agreement with our results, various studies have recently shown the non-association of the rs352140 polymorphism in the *TLR9* gene among different liver diseases. Jungie et al. have reported that there was no significant association between rs352140 genotypes in 211 HCC patients compared to healthy controls [12]. Kikuchi et al. also showed in patients with primary biliary cirrhosis (PBC) that there was no significant association between the genotype frequency of this polymorphism and the PBC risk [13]. While another study by Youssef et al., which examined the association of the rs352140 polymorphism in Egyptian patients with chronic HCV, showed that the distribution frequency of AA genotype was significantly higher in the F1-F2 hepatic fibrosis group compared with the F3-F4 group; also, the A allele may increase the risk of progressive liver fibrosis [14]. It is noteworthy that in this study, the allelic and genotypic frequencies of the study population were not in Hardy-Weinberg equilibrium due to the zero frequency of the GG genotype.

**Table 5:** Genotype distribution of *TLR9* polymorphisms in NAFLD patients with different hepatic fibrosis stages *vs* healthy controls

**Genotypes**		**Healthy controls **	**F0-F1 patients**	**F2-F3 patients**	**F4 patients**
rs352140	GG	6 (28.6)	9 (27.3)	7 (36.8)	5 (27.8)
	GA	11 (52.4)	16 (48.5)	8 (42.1)	11 (61.1)
	AA	4 (19)	8 (24.2)	4 (21.1)	2 (11.1)
					
rs5743836	TT	16 (72.7)	29 (87.9)	13 (68.4)	15 (83.3)
	TC	5 (22.7)	4 (12.1)	6 (31.6)	3 (16.7)
	CC	1 (4.5)	0	0	0
					
rs187084	TT	10 (47.6)	14 (43.8)	5 (27.8)	6 (33.3)
	TC	9 (42.9)	11 (34.4)	9 (50)	9 (50)
	CC	2 (9.5)	7 (21.9)	4 (22.2)	3 (16.7)

In confirmation of our results, Fischer and co-workers showed that there was no significant association between allelic and genotypic frequency of rs5743836 with susceptibility risk to chronic hepatitis C (HCV) in Western Europe [15]. While in the study by Zayed et al. to investigate the association of *TLR9* rs5743836 single nucleotide polymorphism with the risk of hepatic fibrosis in patients with chronic hepatitis C infection showed that the genotype frequency of this polymorphism was significantly different in HCV positive and healthy individuals [9]. 

**Figure 1:**
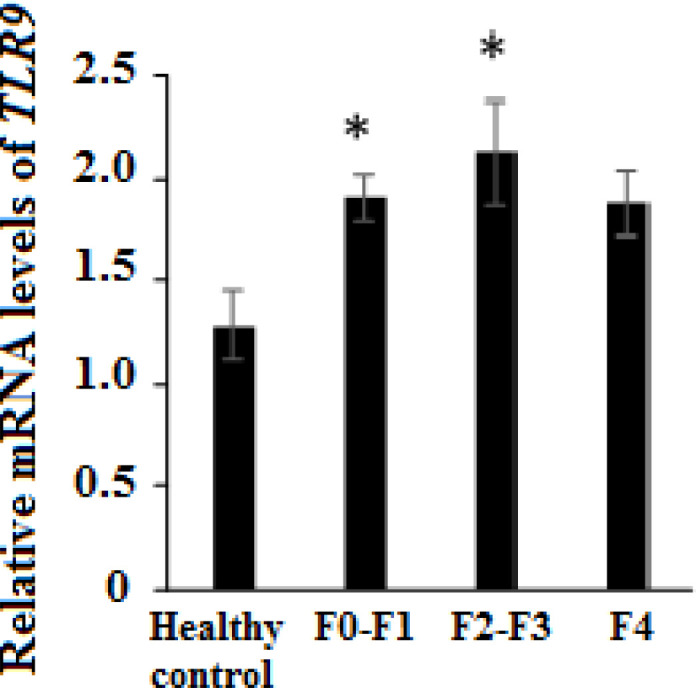
Gene Expression of Toll-Like Receptors 9 in NAFLD patients. Relative mRNA level of *TLR9* in NAFLD patients with F0-F1, F2-F3, and F4 hepatic fibrosis stages compared to healthy controls, was determined using qPCR. Asterisks indicated P<0.05 compared to healthy controls

The analysis of gene expression showed incremented mRNA expression level of the *TLR9* gene in NAFLD patients with F0-F1 (P=0.007) and F2-F3 (P=0.032) liver fibrosis compared with healthy controls. We found no significant overexpression of *TLR9* mRNA in cirrhotic patients (F4 liver fibrosis stage) compared with healthy controls (P=0.10) (Fig. 1). Also, no significant difference was found in the *TLR9* mRNA expression levels in the F2-F3 group compared to the F0-F1 (P=0.80) and F4 (P=0.79) groups.

Pearson correlation analysis was performed to investigate the relationship between the mRNA expression levels of the *TLR9* gene with the studied genetic polymorphisms in NAFLD patients with different degrees of hepatic fibrosis. The results showed a significant correlation between increased risk of fibrosis progression and elevated *TLR9* mRNA levels in NAFLD patients (P=0.016, r=0.25). However, there was no significant correlation between the expression levels of this gene and the genotypes distribution of the rs352140 (P=0.098, r=0.17), rs5743836 (P=0.72, r=0.037) and rs187084 (P=0.15, r=0.15) polymorphisms.

Inconsistent with our results, previous studies have also shown that liver injury and fibrosis induced by carbon tetrachloride, acetaminophen, and bile duct ligation is less in *TLR9*^-/-^ mice, indicating that *TLR9* plays a key role in liver diseases [6, 16]. Miura and coworkers showed that steatohepatitis is significantly reduced in *TLR9*^-/-^, *IL-1βR*^-/-^, and *MyD88*^-/-^ mice treated with choline-deficient amino acid defined (CDAA) diet. Also, they showed that activation of the TLR9 downstream signaling pathway leads to IL-1β production. IL-1β induces lipid accumulation and cell death in hepatocytes and fibrogenic mediators expression in hepatic stellate cells, resulting in steatosis, hepatocyte injury, and fibrosis [6].

Garcia-Martinez et al. also have shown that the level of hepatocyte-derived mitochondrial DNA is elevated in plasma samples obtained from mice and NASH patients, and it has the ability to TLR9 activation [17]. They also showed that the presence of TLR9 on lysozyme-expressing cells is necessary for the development of NASH in response to a high-fat diet and a TLR9 antagonist prevented the development of NASH [17]. These data confirm that TLR9 pathway activation provides a link between the critical metabolic and inflammatory phenotypes in NASH. Our results also showed an increment overexpression of *TLR9* gene mRNA in F0-F1 and F2-F3 patients compared to healthy control which is correlated with liver fibrosis progression from F0-F1 to F2-F3.

In confirmation of no significant increase in mRNA levels of *TLR9* in F4 patients compared with healthy individuals, previous studies have shown that during the progression of fibrosis from F1 to F2, the severity of inflammation in the hepatic lobules increases. However, with the progression of fibrosis to higher degrees and in the stage of cirrhosis, the severity of steatosis and necroinflammatory reactions in patients will return to normal, which is called NASH melting [18, 19].

As a preliminary study, given the limitations of the present study to collect more patients with liver fibrosis, a significant relationship between the elevated mRNA levels of *TLR9* with liver fibrosis progression was found in NAFLD patients; while the association between studied polymorphisms and the risk of developing liver fibrosis caused by NAFLD was not observed.

## Conflict of Interest:

The authors have no relevant financial or non-financial interests to disclose.
